# What is epidemiology? Changing definitions of epidemiology 1978-2017

**DOI:** 10.1371/journal.pone.0208442

**Published:** 2018-12-10

**Authors:** Mathilde Frérot, Annick Lefebvre, Simon Aho, Patrick Callier, Karine Astruc, Ludwig Serge Aho Glélé

**Affiliations:** 1 Department of Hospital Epidemiology and Infection Control, Dijon University Hospital, Dijon, France; 2 Department of Hospital Epidemiology and Infection Control, Reims University Hospital, Reims, France; 3 Department of Medical Oncology, Lorraine Institute of Oncology, Nancy, France; 4 Department of human genetics, Dijon University Hospital, Dijon, France; Monash University, AUSTRALIA

## Abstract

**Context:**

Epidemiology is a discipline which has evolved with the changes taking place in society and the emergence of new diseases and new discipline related to epidemiology. With these evolutions, it is important to understand epidemiology and to analyse the evolution of content of definitions of epidemiology.

**Objectives:**

The main objective of this paper was to identify new definitions of epidemiology available since 1978. Secondary objectives were to analyse the content of these definitions, to compare them with those used by Lilienfeld and to determine whether changes have taken place over the last forty years.

**Methods:**

A review of grey literature and published literature was conducted to find the definitions of epidemiology written between 1978 and 2017.

**Results:**

102 definitions of epidemiology were retained. They helped to highlight 20 terms and concepts related to epidemiology. Most of them were already used in the definitions used by Lilienfeld. Five terms were present in more than 50% of definitions from the period 1978 to 2017: “population”, “study”, “disease”, “health” and “distribution”. Several developments have occurred: strengthening of the terms “control” and “health” already used, the concept of “disease” was less frequently encountered whereas the concepts “infectious diseases”, “mass phenomenon” are no longer used in definitions from 1978 to 2017.

**Conclusion:**

This evolution of content of definition of epidemiology is absent from books on epidemiology. A thematic analysis of definitions of epidemiology could be conducted in order to improve our understanding of changes observed.

## Introduction

Epidemiology is a recent discipline which has evolved with the changes taking place in society and the emergence of new diseases. This evolution has allowed epidemiology to remain a useful and relevant tool in bringing to light and understanding diseases and health events. Since its origins, more than a century ago, many definitions of epidemiology have been suggested.

In 1978, Lilienfeld published a seminal paper on definitions of epidemiology. For him, there was no consensus among epidemiologists as to the definition of epidemiology. The objective of his work was to provide a single and understandable definition of epidemiology that was suitable for all types of diseases and populations. Lilienfeld based his work on 23 existing definitions of epidemiology and proposed the following definition [1]: "*Epidemiology is a method of reasoning about disease that deals with biological inference derived from observations of disease phenomena in population groups*".

The publication of this article resulted in many comments, including three letters to the Editor [2] each discussing the limits of this new definition. In his letter, Evans analyzed the content of the 23 definitions used reviewed by Lilienfeld. He listed the various terms and concepts used in these definitions and determined their frequency of use. After this work, he proposed a different definition of epidemiology [3]: "*Epidemiology is the quantitative analysis of the circumstances under which disease processes*, *including trauma*, *occur in population groups*, *factors affecting their incidence*, *distribution*, *and the host response and use of this knowledge in prevention and control* ".

Since the work of Lilienfeld and Evans, new definitions of epidemiology have been proposed. During this time, many fields related to epidemiology (pharmacoepidemiology, molecular epidemiology, genetic epidemiology…) have expanded. Moreover, epidemiology is related to many disciplines such as ethics [4], philosophy [5] and epistemology [6]. These disciplines used existing definitions of epidemiology. Broadbend [5], in his work on philosophy and epidemiology, used the definition of epidemiology of Rothman and Last (p23) and insisted on the notion of comparisons of groups as did Morabia [7]. Given the evolution in fields related to epidemiology, it is important to understand epidemiology and to analyse the evolution of definitions of epidemiology.

The main objective of this paper was to identify new definitions of epidemiology available since 1978, including veterinary medicine and epidemiology subspecialties [7, 8]. Secondary objectives were to analyse the content of these definitions, to compare them with those used by Lilienfeld and to determine whether changes have taken place over the last forty years.

## Materials and methods

### Literature review

A review of grey literature and published literature was conducted to find definitions of epidemiology written between 1978 and 2017. It was conducted in English.

Grey literature on the subject was retrieved using the search engine "Google scholar” with the following keywords: (*Definition* AND *Epidemiology*) OR (*definition of epidemiology*). Definitions of epidemiology included in books were retrieved via Google books (https://books.google.fr/) and Amazon (http://www.amazon.fr/), Library of Congress (https://catalog.loc.gov/), The British Library (http://explore.bl.uk/primo_library/libweb/action/search.do?vid=BLVU1#) and NLM Catalog (https://www.ncbi.nlm.nih.gov/nlmcatalog/). Among the epidemiology textbooks identified, the book “Epidemiology and the people’s health: theory and context” by Nancy Krieger [9] contains a table with definitions of epidemiology from 1922 to 2007 retrieved from epidemiology textbooks. Works of Bhopal [10] and Zhang [11] were also considered as starting point. These definitions were selected as starting point for our work.

Definitions in the published literature were retrieved from "PubMed" and "ScienceDirect" using the following keywords: (*Definition* AND *Epidemiology)* OR (*Definition of epidemiology*) and the names of authors identified during the search of the grey literature.

Definitions for which the author could not be identified, those prior to 1978, those cited in Lilienfeld’s article [1] or those that could be related to the definition proposed by Lilienfeld or another author were eliminated.

Definitions provided by online dictionary sites, encyclopaedias like Wikipedia or internet sites dedicated to students were also retained, using the strategy mentioned above (keywords: (Definition AND Epidemiology) OR (definition of epidemiology)).

### Analysis of the content of the new definitions

The content of new definitions of epidemiology was analyzed to identify the terms and concepts present in these definitions. Once a term or concept was identified in at least two definitions, it was added to the list of terms.

### Analysis of the evolution of definition content between 1978 and 2017

In 1979, Evans [3] analyzed the content of the definitions used by Lilienfeld to build his definition of epidemiology. In summary, he identified 23 different terms and concepts. For each term and concept, the frequency of occurrence in the definitions was calculated. Then, they were grouped into eight categories: status of the person (disease, infectious disease, physiologic conditions, injuries, health); group affected (populations, community, mass phenomena, outbreak); distribution of disease; spread (spread, propagation, dynamics); incidence, occurrence; etiology (causes, determinant factors, circumstances of occurrence, ecology); understanding disease (natural history or nature, understanding the process); prevention and control.

To study the evolution of the content of definitions of epidemiology over time, the content of the definitions used by Lilienfeld was compared with new content from the period 1978–2017.

For each term and concept identified by Evans, the frequency of appearance in the definitions from the period 1978–2014 was estimated.

For each term and concept identified using the definitions from 1978 to 2017, the frequency of appearance in the definitions used by Lilienfeld was estimated.

Then for each term and concept, whatever the period, the frequency of appearance in the definitions used by Lilienfeld and definitions from 1978 to 2017 were compared.

All statistical comparisons were performed using bayes factors [12] with R software [13] (package BayesFactor). We have chosen bayes factor instead of p value, which is used in the context of null-hypothesis significance testing (NHST). Indeed, a nonsignificant p-value does not quantify evidence in favour of the null hypothesis. As stated by the American Statistical Association, “a relatively large p-value does not imply evidence in favor of the null hypothesis” [14].

## Results

### Literature search

The search of published and grey literature revealed 102 definitions of epidemiology: 93 for human medicine, including subspecialties of epidemiology (n = 24) and 9 for veterinary medicine. We have selected 29 definitions of epidemiology from websites. The definitions of epidemiology were found in different media: articles, epidemiology online courses and excerpts from books. A total of 69 definitions from 1978 to 2017 were selected (Table 1).

**Table 1:** Definitions of epidemiology from the period 1978–2017.

AUTHOR (Reference)	DATE	DEFINITION
**Frerichs RR et al. [33]**	1978	Epidemiology is the study of the prevalence and dynamics of stages of health in populations
**Barker DJP et al. [102]**	1979	Epidemiology, the study of the distribution and determinants of disease in human populations, has always been an integral part of medical practice
**Cole P [82]**	1979	Epidemiology is the science dealing with the environmental causes of diseases of humans as inferred from observations of human beings
**Rich H [38]**	1979	Epidemiology is the science of the dynamics of health in populations
**Evans AS [3]**	1979	Epidemiology is the quantitative analysis of the circumstances under which disease processes, including trauma, occur in population groups, the factors affecting their incidence, distribution, and host responses, and the use of this knowledge in prevention and control
**Lilienfeld A et al. [103]**	1980	Epidemiology is concerned with the patterns of disease occurrence in human populations and the factors that influence these patterns
**Kleinbaum DG et al. [27]**	1982	Epidemiology is the study of health and illness in human populations
**Miettinen OS [104]**	1985	The discipline of epidemiology is “the aggregate of principles of studying the occurrence of illness and related states and events”
**Weiss NS [105]**	1986	Epidemiology per se is the study of variation in the occurrence of disease, and the reasons for that variation
**Kelsey JL et al. [25]**	1986	Epidemiology, the study of the occurrence and distribution of disease and other health -related conditions in populations, is used for many purposes
**Shy CM [83]**	1986	Epidemiology is a study of the occurrence and distribution of disease in populations and the factors that account for this distribution
**Hennekens CH et al. [106]**	1987	The study of the distribution and determinants of disease frequency' in human populations
**Buck C [15]**	1988	Besides its importance and usefulness in disease surveillance and prevention, epidemiology has an even more critical function to carry out- the gathering of knowledge for understanding the health-disease process. It can anticipate needs, identify risk conditions, and orient the definition of priorities and the use of available resources for planning and administering health systems. In short, by analyzing and evaluating health problems and health services, and their contexts, epidemiology can go beyond considering just specific health problems: it can help bring us closer to considering society as the source for explaining health problems and their solutions
**Ahlbom A et al. [107]**	1990	Epidemiology is the science of occurrence of diseases in human populations
**Kuller LH [80]**	1991	Epidemiology is the study of “epidemics” and their prevention
**Vaughan P et al. [28]**	1991	Epidemiology can be considered as the study of the distribution of problems related to health and disease and their determinants in human populations. The purpose of epidemiology is to collect, interpret and use information to promote health and reduce disease
**Kelsey JL [108]**	1996	Same definition as Kelsey, 1986
**Berkman LF et al. [34]**	1997	Epidemiology is the study of the distribution and determinants of states of health in populations
**Savitz DA et al. [19]**	1999	In defining epidemiology, some note that the principal applications of epidemiologic knowledge are to disease prevention and control, but all refer to some variant of “the study of disease”, thus defining epidemiology as a science
**Schoenbach V et al.[16]**	1999	Well, some epidemiologists study the skin. But epidemiologists study all kinds of diseases and other aspects of health, also. Epidemiology is the study of health and disease in populations. It’s a basic science of Public Heath
**Schwartz S et al. [41]**	1999	Epidemiology is a discipline dedicated to understanding the causes of health states in population
**Vetter NJ et al. [109]**	1999	Epidemiology is the study of the distribution and change in diseases. The purpose of epidemiology is to identify things in people and their surroundings that affect the occurrence of disease
**Moon G et al. [74]**	2000	Quoting Lilienfeld, 1980 [103]
**Last JM [43]**	2001	The study of the distribution and determinants of health-related states or events in specified populations, and the application of this study to control of health problems
**Timmreck TC [110]**	2002	Epidemiology is an investigative method used to detect the cause or source of diseases, disorders, syndromes, conditions, or perils that cause pain, injury, illness, disability, or death in human populations or groups. … Epidemiology has been defined in several ways. One definition is the study of the nature, cause, control, and determinants of the frequency and distribution of disease, disability, and death in human populations.
**Friedman G et al.[111]**	2003	Epidemiology is the study of disease occurrence in human populations. The primary units of concern are groups of persons, not separate individuals.
**Gerstman BB [30]**	2003	Modern definitions of epidemiology refer to distributions in populations (statistical), determinants of health and disease (pathophysiological, environmental, behavioral), control of health problems (biological, social, economic, political, administrative, legal)
**Coggon D et al. [112]**	2003	Epidemiology is the study of how often diseases occur in different groups of people and why. Epidemiological information is used to plan and evaluate strategies to prevent illness and as a guide to the management of patients in whom disease has already developed
**Farmer RDT et al. [113]**	2004	…In contrast to clinical medicine, epidemiology involves the study of groups of people (populations) rather than direct study of individuals
**Friis RH et al. [29]**	2004	Epidemiology is concerned with the distribution and determinants of health and diseases, morbidity, injuries, disability, and mortality in populations
**Morabia A [40]**	2004	The blending of population thinking and group comparisons in an integrated theory to appraise health-related causal relationships characterizes epidemiology
**Rossignol A [20]**	2005	Epidemiology is the foundational science of public health. Much as a yardstick measures length, epidemiologic investigations measure and compare the frequencies of disease, injury, and other health-related events in human populations
**Stedman TL [39]**	2005	Epidemiology is defined as the study of the distribution and determinants of health related states or events in human populations and the application of this study to the prevention and the control of health problems
**Oakes JM et al. [36]**	2006	Epidemiology is the study of the distribution and determinants of states of health in populations
**Yarnell J [52]**	2007	At the beginning of the twenty-first century, epidemiology is a broad-based population science, drawing on many disciplines from biology and sociology to biostatistics and philosophy of science, which investigates the causes of human disease and methods for their control
**Boslaugh S [53]**	2008	Epidemiology is the study of frequency and determinants of morbidity and mortality in populations
**Bhopal RS [17]**	2008	Epidemiology is the science and craft that studies the pattern of disease (and health, though usually indirectly) in populations to help understand both their causes and the burden they impose. This information is applied to prevent, control or manage the problems under study
**Porta M [42]**	2008	The study of the occurrence and distribution of health-related states or events in specified populations, including the study of the determinants influencing such states, and the application of this knowledge to control health problems
**Gordis L [114]**	2009	Epidemiology is the study of how disease is distributed in populations and the factors that influence or determine this distribution
**Olsen J et al. [115]**	2010	Epidemiology is defined by the object of research, “to identify determinants that change the occurrence of health phenomena in human populations.”
**Saracci R [21]**	2010	“Epidemiology is the study of health and disease in populations”. The population aspect is the distinctive trait of epidemiology, while health and disease are investigated at other levels as well.
**Webb P et al. [35]**	2010	Epidemiology … is about measuring health, identifying the causes of ill-health, and intervening to improve health… Perhaps epidemiology's most fundamental role is to provide a logic and structure for the analyses of health problems both great and small
**Carneiro I et al. [51]**	2011	Epidemiology is the study of the distribution and determinants of health states or events in specified populations, and the application of this study to control of health problems (Adapted from Porta, 2008). Quoted reference: [116]
**Fos PJ [44]**	2011	Epidemiology is the study of the distribution and determinants of health-related states and events in defined populations and the application of this study to the control of health problems (Adapted from Last, 2001) Quoted reference: [43] The Greek root of epidemiology and the two definitions have a common theme, namely, the people. The people are considered as a group, which is referred to as a population. This population-centered nature of epidemiology leads to one of the differences between public health services and medical services. … Epidemiology is the study of factors affecting the health and illness of populations.
**Krickeberg K et al. [117]**	2011	…epidemiology is about illness in populations, not in individual subjects. More precisely, it is concerned with the frequency of diseases in various parts of the population in which we are interested, the so-called "target population".
**Rothman KJ [18]**	2012	Often considered the core science of public health, epidemiology involves “the study of the distribution and determinants of disease frequency", or put even more simply "the study of the occurrence of illness
**Ward H et al. [22]**	2012	Epidemiology (is) the study of the distribution and determinants of health and illness in populations.
**Aschengrau A et al. [26]**	2013	The study of the distribution and determinants of disease frequency in human populations and the application of this study to control health problems"
**Ahrens W et al. [45]**	2013	Epidemiology is the study of the distribution and determinants of disease frequency (quoting MacMahon,1970) Epidemiology is the study of the distribution and determinants of health-related states or events in specified populations, and the application of the study to control of health problems (Quoting Last, 2001) Quoted references: [43, 69]
**Broeck JV et al. [118]**	2013	According to the broadest of views, epidemiology is synonym of community medicine (Miettinen 2011a, b) Quoted references: [119, 120]
**Gerstman BB [121]**	2013	Quoting several authors and quoting Last, 2001 [43]
**Macera CA et al. [46]**	2013	Epidemiology is the study of a scientific method of problem solving that helps “disease detectives” understand how people get sick and die, who gets sick and die, and how to avoid getting sick. The current definition of epidemiology is the study of the distribution (who has the problem) and determinants (things that influence the problem) of health-related conditions in human populations and the application of this method to the control of health problem.
**Yarnell J et al. [122]**	2013	Same definition as Yarnell, 2007
**Fletcher RH et al. [123]**	2014	Epidemiology is the study of disease occurrence in human populations by counting health related events in people in relation to the naturally accruing groups (populations) of which they are members
**Khan AR et al. [47]**	2014	The International Epidemiological Association defines epidemiology as “the study of the distribution and determinants of health related states and events in the populations and the application of this study to the control of health problems”
**Katz DI et al. [124]**	2014	Epidemiology is usually defined as the study of factors that determine the occurrence and distribution of disease in population
**Keyes KM et al. [31]**	2014	Epidemiology is the science of understanding the causes and distribution of population health so that we may intervene to prevent disease and promote health
**Szklo M et al. [37] [33]**	2014	Epidemiology is traditionally defined as the study of the distribution and determinants of health-related states or events in specified populations and the application of this study to the control of health problems
**Weiss NS et al. [125]**	2014	Epidemiology concerns describing and understanding patterns of disease occurrence in human populations, with the ultimate goal of preventing disease
**Merrill RM [48]**	2015	Epidemiology is the study of the distribution and determinants of health-related states or events in human population and the application of this study to the prevention and control of health problems (Stedman’s dictionary. 5th edition, 2005) Quoted reference: [39]
**Patten S [126]**	2015	Epidemiology is the study of the distribution and determinants of disease in populations
**Schneider D et al. [49]**	2015	Epidemiology is "The study of the occurrence and distribution of health-related events, states, and processes in specified populations, including the study of the determinants influencing such processes, and the application of this knowledge to control relevant health problems." (Adapted from Lilienfeld, 1980). Quoted reference: [103]
**Wassertheil-Smoller S et al. [23]**	2015	Epidemiology may be defined as the study of the distribution of health and disease in groups of people and the study of the factors that influence this distribution. Modern epidemiology also encompasses the evaluation of diagnostic and therapeutic modalities and the delivery of health-care services.
**Bhopal RS [50]**	2016	…epidemiology will be defined here as the science and practice that describes and explain disease patterns in populations. It uses this knowledge to prevent and control disease, and improve health. The central idea of epidemiology is that patterns of ill health and disease in population may be analyzed systematically to understand their causes and to improve health.
**Stewart A [127]**	2016	Epidemiology is the study of how often diseases occur in different groups of people, and why (quoting Coggon et al., 1997). Note: same definition of epidemiology as Coggon, 2003 (5^th^ edition) and Coggon, 1997 (4^th^ edition)
**Webb P et al. [128]**	2016	Same definition as Webb, 2010
**Friis RH [129]**	2017	Same definition as Friis, 2004
**World Health Organization [32] (WHO) [22]**	No information	Epidemiology is the study of the distribution and determinants of health-related states or events (including disease), and the application of this study to the control of diseases and other health problems. Various methods can be used to carry out epidemiological investigations: surveillance and descriptive studies can be used to study distribution; analytical studies are used to study determinants

Analysis of the content of definitions from the period 1978 to 2017

### Human medicine, general epidemiology

A total of 20 terms and concepts were identified in definitions from 1978 to 2017. Of the 20 terms and concepts, only three were not present in the definitions used by Lilienfeld: "*knowledge*" appeared in 1979 [3], "*problems*" appeared in 1991 [15] and “*public health*” appeared in 1999 [16] Table 2 shows the frequencies of appearance of terms in definitions from 1978 to 2017.

**Table 2:** Frequency of occurrence of the terms identified from 69 definitions from the period 1978–2017.

Terms used*	Definition using terms	
	Number	%
Population	58	84
Disease/Illness	51	74
To study/Studying	47	68
Health	40	58
Distribution	34	49
To determine/ Determinant	30	43
States/Stages/Events/Conditions	34	49
To occur/Occurrence	19	28
Control	22	32
Problems	20	29
Science	12	17
Human populations/Human groups	17	25
To prevent/ Prevention	11	16
Factors	8	12
Knowledge	6	9
To understand/ understanding	7	10
Frequency	7	10
Dynamics	2	3
Process	3	4
Public health	4	6

* “The terms examined by Lilienfeld have been grouped, in the spirit of his article, into categories”.

The term “*population*” or “*group*” was found in 84% of the definitions (58/69). For 25% of definitions (17/69), it was stated that epidemiology applies to human beings.

For 68% of the definitions (47/69), epidemiology was "*the study of something*" (study of …) and 17% of the definitions (12/69) defined epidemiology as a “*science*”. Four definitions (11%) used both terms [16–19].

Epidemiology was associated with “*health*” in 58% of definitions (40/69) and “*disease*” in 74% of definitions (51/69). Both terms were present in 23% of the definitions (16/69) [15–17, 20–32]. In 25% (17/69) of definitions, the term “disease” was not present but the terms “health problem” or “health states” was present [33–42] [43–49].

The concept of “*health control*” or “*disease control*” was present in 32% of the definitions (22/69). The concept of “*disease prevention”* or “prevention of *health problems*” was present in 16% of the definitions (11/69). These notions were associated in 9% (6/69) of the definitions [3, 17, 19, 39] [48, 50].

The term “*problem*” was present in 29% of the definitions (20/69). In 25% of definitions (17/69), the terms “*problem*” and “*health*” were associated (“health *problem*”) [15, 26] [28, 30, 32, 35, 37, 39, 42–49, 51].

Epidemiology was associated with the field of statistics in 6% (4/69) of the definitions [3, 30, 32, 52].

Analysis of the evolution of definition content between 1978 and 2017

Table 3 shows a comparison of the content of definitions reviewed by Lilienfeld and those from 1978 to 2017 according to the concepts identified by Evans.

**Table 3:** Comparison between the contents of definitions reviewed by Lilienfeld and definitions from the period 1978–2017 according to the terms and concepts identified by Evans [3].

Terms	Definition from the period 1978–2017 (N = 69)		Definition reviewed by Lilienfeld (N = 23)		BF*
	Number	%	Number	%	
Statut of person					
Diseased (see response 2.6)	51	74	21	91	1.1
Infectious disease	0	0	4	17	65.9
Physiological condition	0	0	1	4	2
Injuries	3	4	1	4	0.5
Health	40	58	1	4	18709.8
Group affected					
Population	58	84	11	48	63.1
Community	1	1	3	13	4.8
Mass phenomena/ Outbreak	0	0	3	13	18.3
Total with group affected mentioned	58	84	17	74	0.5
Distribution of disease	14	20	9	39	1.2
Spread (propagation. spread. dynamics)	2	3	3	13	2.1
Incidence. Occurrence	20	29	5	22	0.3
Etiology					
Causes. Determinant. Factors	28	41	5	22	0.8
Circumstances of occurrence	1	1	2	9	1.9
Ecology	0	0	1	4	2
Total with etiology mentioned	29	42	8	35	0.3
Understanding disease					
Natural history or nature	1	1	2	9	1.9
Understanding the process	7	10	1	4	0.5
Total with understanding disease mentioned	3	4	3	13	1.2
Prevention and Control	6	9	3	13	0.5

* BF: Bayes Factor

Several concepts (“*infectious disease*”, “*mass phenomena*”…) identified by Evans were not present in definitions from 1978 to 2017 (Table 3).

The term “*health*” was more frequent in definitions from 1978 to 2017 than in the definitions used by Lilienfeld (58% versus 4%, Bayes Factor(BF) = 18709.9).

The term “infectious disease” was less frequent in definitions from 1978 to 2017 than in the definitions used by Lilienfeld, with a BF estimated to 65.9 (0% versus 17%). "Mass phenomena/outbreak" and “infectious disease” can be related. The BF associated with "mass phenomena/outbreak, was estimated at 18, and this also reflects a decrease in the use of the term “infectious disease”.

The term “*population*” was more frequent in definitions from 1978 to 2017 than in the definitions used by Lilienfeld (84% versus 48%, Bayes (BF) = 63.1).

Table 4 shows a comparison of the content of definitions used by Lilienfeld and those from 1978 to 2017 according to the terms and concepts we have identified.

**Table 4:** Comparison between the content of definitions used by Lilienfeld and definitions from the period 1978 to 2017 according to the terms and concepts identified in definitions from the period 1978–2017.

Terms	Definition from the period 1978–2017 (N = 69)		Definition used by Lilienfeld (N = 23)		BF*
	Number	%	Number	%	
Population	58	84	11	48	63.1
Disease/Illness	51	74	21	91	1.2
To study/Studying	47	68	14	61	0.3
Health	40	58	1	4	18709.8
Distribution	34	49	9	39	0.3
To determine/ Determinant	30	43	5	22	1.2
States/Stages/Events/Conditions	34	49	3	13	31.3
To occur/Occurrence	19	28	3	13	0.6
Control	22	32	1	4	11.5
Problems	20	29	0	0	43.8
Science	12	17	5	22	0.3
Human populations/Human groups	17	25	7	30	0.3
To prevent/ Prevention	11	16	2	9	0.4
Factors	8	12	5	22	0.7
Knowledge	6	9	0	0	0.9
Frequency	7	10	1	4	0.5
To understand/ Understanding	7	10	1	4	0.5
Dynamics	2	3	1	4	0.6
Process	3	4	1	4	0.5
Public health	4	6	0	0	0.7

* BF: Bayes Factor

For the term “*population*” and “*health*”, the results shown in Table 3 and Table 4 are identical.

The term “*problems*” was probably more frequent in definitions from 1978 to 2017 than in the definitions used by Lilienfeld (29% versus 0%, BF = 43.8).

### Human medicine, subspecialities of epidemiology

The following subspecialties (outcome-oriented or exposition-oriented) were taken into account, according to Boslaugh [53], Rothman [24]and Carter-Pokras, [8], among many others: cancer epidemiology; cardiovascular epidemiology; obesity epidemiology; nutritional epidemiology; psychiatric epidemiology; genetic and molecular epidemiology; molecular epidemiology; genetic epidemiology; infectious diseases epidemiology; reproductive and perinatal epidemiology; environmental epidemiology; occupational epidemiology; social epidemiology. A total of 24 definitions were selected (Table 5).

**Table 5:** Selected definitions of epidemiology, by subspecialty.

AUTHOR (Reference)	DATE	DEFINITION
**Dos Santos Silva I [64]** Cancer	1999	“Epidemiology is the study of the distribution and determinants of health-related states or events in specified populations, and the application of this study to control of health problems.” Quoting Last, 1995 [62] Cancer epidemiology is the branch of epidemiology concerned with the disease cancer. Therefore, this definition is as valid to cancer epidemiology as it is to epidemiology in general.
**Schottenfeld D et al. [60]** Cancer	2006	No definition of epidemiology provided
**Adami HO et al. [130]** Cancer	2008	No definition of epidemiology provided The roles of epidemiology have been to describe the distribution of cancer in populations and to seek deviations from randomness that offer clues as to their explanations, to assess the validity and strength of associations that are suspected causes, and to evaluate the effectiveness of preventive measures by continued monitoring of cancer incidence and mortality.
**Loda M et al. [131]** Cancer	2016	According to the World health Organization (WHO), epidemiology is "the study of the distribution and determinants of health-related states of events. and the application of this study to the control of diseases and other health problems" Quoting WHO, 2014 http://www.who.int/topics/epidemiology/en/
**Luepker RV [84]** Cardiovascular	2009	Epidemiology is the study of disease patterns and outcomes in populations. Because of this public health perspective, epidemiologists also play a learning role in the study of disease prevention. Observational epidemiologists define the role of risk factors in predicting, preventing, and treating cardiovascular disease through population studies. Working with laboratory medical and behavioral scientists, they elucidate the underlying mechanisms and initiate large prevention and treatment trials. Our understanding of lipids, hypertension, smoking, diabetes, inflammation, and other factors leading to cardiovascular diseases originated in epidemiological observations.
**Labarthe DR [59]** Cardiovascular	2010	No definition of epidemiology provided
**Hu F [54]** Obesity Epidemiology	2008	No definition of epidemiology provided, but referring to classic textbooks in epidemiology: Morris, 1964 [132]; MacMahon, 1970 [69], Lilienfeld, 1976 [133], Miettinen, 1985 [104]; Rothman, 1986 [134]. Obesity epidemiology uses epidemiologic approaches to examine the causes and consequences of obesity in human populations. It includes the study of multiple, broad, interrelated domains such as (a) the distributions, patterns, and dynamics of obesity in populations; (b) health and other consequences of obesity: (c) the determinants or causes of obesity; and (c) the development and validation of body composition measurement methods used in epidemiologic studies. Knowledge gained is eventually applied to public health initiatives to prevent and control obesity and related health conditions.
**[55]** Nutritional Epidemiology	2012	No definition of epidemiology provided, but recommended books. Although I have not attempted to define or explain basic epidemiologic terms, most of the chapters can be read by someone with elementary statistical knowledge and some epidemiologic background. Readers without exposure to epidemiology would profit by referring to an introductory text such as MacMahon and Trichopoulos’ Principles of Epidemiology, Aschengrau and Seage’s Essentials of Epidemiology in Public Health, Rothman’s Epidemiology: An Introduction, or Rothman, Greenland, and Lash’s Modern Epidemiology, the last being the most advanced. Similarly, epidemiologists without formal exposure to nutrition can benefit by reading… Quoted books: [18, 24, 26, 135] The field of nutritional epidemiology has developed from interest in the concept that aspects of diet may influence the occurrence of human disease.
**Susser E et al. [61]** Psychiatry	2006	No definition of epidemiology provided
**Tsuang MT et al. [65]** Psychiatry	2011	Quoting Last, 2001 [43]; Wikipedia ; Rothman, 2008 [24] Epidemiology, according to Last’s Dictionary of Epidemiology, is ‘The study of the distribution and determinants of health-related states or events in specified populations and the application of this study to control of health problems’ Wikipedia states ‘Epidemiology is the study of factors affecting the health and illness of populations, and serves as the foundation of interventions made in the interest of public health and preventive medicine’ Rothman and Greenland: ‘the ultimate goal of most epidemiologic research is the elaboration of causes that can explain patterns of disease occurrence’ Psychiatric epidemiology is simply the epidemiology of psychiatric disorders–no more, no less. The principles and practice are the same when studying psychiatric disorder as they are when studying other medical conditions
**Ioannidis JPA [56]** Genetic and molecular epidemiology	2007	No definition of epidemiology provided Genetic and molecular epidemiology, the investigation of genetic and molecular determinants of health and disease…
**Schulte PA et al. [57]** Molecular Epidemiology	2012	No definition of epidemiology provided Progress in the molecular approach to biology and medicine has simulated and excited both the public and researchers, who now believe these advances can be applied to the study, prevention and control of health risks faced by human populations. The term “molecular epidemiology” may be used to describe such an approach: the incorporation of molecular, cellular, and other biologic measurements into epidemiological research.
**Burton PR et al. [66]** Genetic epidemiology	2005	Epidemiology is usually defined as “the study of the distribution, determinants [and control] of health-related states and events in populations”. Quoting Last, 2001 [43] By contrast, genetic epidemiology means different things to different people (referring six authors) We regard it as a discipline closely allied to traditional epidemiology that focuses on the familial, and in particular genetic, determinants of disease and the joint effects of genes and non-genetic determinants.
**Palmer L et al. [67]** Genetic epidemiology	2011	Quoting Last, 2001 [43] and Burton, 2005 [66]
**Austin M et al. [58]** Genetic epidemiology	2013	No definition of epidemiology provided " … an emerging field … that represents an important interaction between the two parent disciplines: genetics and epidemiology. Genetic epidemiology differs from epidemiology by its explicit consideration of genetic factors and family resemblance; it differs from population genetics by its focus on disease; it also differs from medical genetics by its emphasis on population aspects." Quoting Rao, 1984 [90]
**Teare MD [136]** Genetic epidemiology	2016	Genetic epidemiology. Is the study of the role of genes and environments on markers of health and disease risk in population.
**Giesecke J [137]** Infectious diseases	2017	Most standard definitions of epidemiology mention something like “the study of distribution and determinants of diseases in the population”… My own practical view is that epidemiology is about putting people into groups. …Epidemiology identifies such groups, ignoring the uniqueness of its members, and tries to discover whether this division of people into groups tells us something more than we could have learnt by just observing each person separately.
**Louis GMB et al. [68]** Reproductive and Perinatal Epidemiology	2011	Quoting Last, 2001 [43] … the Faculty defines reproductive and perinatal epidemiology for the purposes of this book as the study of the distribution, determinants, and sequelae of reproductive and/or perinatal processes and events. This definition is consistent with the more general definition of epidemiology as defined by John Last (2001) in his textbook A Dictionary of Epidemiology. … the field is essentially defined by reproductive and perinatal outcomes (and not exposures, per se)…
**Wilkinson P [138]** Environmental Epidemiology	2006	No definition of epidemiology provided
**Merrill RM [139]** Environmental Epidemiology	2008	In a book compiled by Last the definition of epidemiology provided is "the study of the distribution and determinants of health-related states or events in specified populations, and the application of this study to control for health problems." Quoting Last, 1995 [62] … epidemiology involves more than just the study of disease states (e.g. respiratory illness), but also includes the study of events (e.g., injury) and behaviors and conditions associated with health (e.g., hand washing). In addition, note that epidemiology is concerned with health-related states or events that occur in populations, not a specific individual Environmental epidemiologists study the frequency and pattern of disease and health-related events and attempt to explain the environmental factors that influence these conditions. The study of why and how environmental factors affect peoples' health is the essence of environmental epidemiology. Environmental epidemiology emphasizes the idea that health is largely influenced by environmental factors, and that by identifying these factors and their modes of transmission, the public's health can be better protected.
**Baker D [140]** Environmental Epidemiology	2008	Environmental epidemiology is a subspecialty of epidemiology, the basic science of public health. Epidemiology is the study of the distribution of health and disease in the population, and of the determinants of this distribution. Environmental epidemiology studies the effects of environmental exposures on health and disease in the population. The subject matter of environmental epidemiology is environmental health: this, in principle, covers all factors external to the human body which may affect health. However, … environmental epidemiology does not try to cover all external factors which may conceivably influence population health, but focuses on physical, chemical and (noninfectious) biological factors in our everyday environment
**Venables K [141]** Occupational epidemiology	2013	Occupational epidemiology is the study of the distribution and determinants of illness and injury related to the work environment. … Furthermore, harmful work exposures can be conceptualized as a “natural experiment” in the causation of illness and injury, because the workplace usually has much better-defined levels and timing of exposure than in the general community.
**O’Campo P et al. [142]** Social epidemiology	2011	Epidemiology is the study of the patterns of health and illness in populations, while social epidemiology focuses on the social determinants that shape the risk and occurrence of poor health in these populations. Quoting Berkman and Kawachi, 2000 [143] and James, 2009 [144].
**Berkman LF et al. [145]** Social epidemiology	2014	Epidemiology is the study of the distribution and determinants of states of health in population. Quoting Susser, 1973 [63]. We define social epidemiology as the branch of epidemiology that studies the social distribution and social determinants of states of health. …We focus on specific social phenomena such as socioeconomic stratification, social networks, discrimination, workplace organization, and public policies rather than on specific disease outcomes.
		Emerging subspecialties
**Brown SL** [96] Medical device epidemiology	2007	Epidemiology, known as the basic science of public health, aims to study the distribution and determinants of diseases in populations …when applied to medical devices, epidemiology may describe patterns of use or factors associated with use or characterize the risk for certain outcomes in defined subgroups.
**Shatin D et al.** [97] Implanted medical device epidemiology	2006	Epidemiology, in general, is “the science of occurrence of diseases in human populations. Disease occurrence is measured and related to different characteristics of individuals or their environments”. Although clinical trials are sometimes considered a subset of epidemiology, we restrict our discussion here to observational studies of patients who receive their implants as a result of routine care. The epidemiology of implanted devices includes the following: (1) Descriptions of patients with implants in terms of demographics, underlying disease, and concurrent disease. (2) Assessment of efficacy and safety of the implant. (3) Comparison of treatments.
**Salathé M** [94] Digital epidemiology	2018	The goal of epidemiology, very broadly speaking, is to understand the patterns of disease and health dynamics in populations as well as the causes of these patterns, and to use this understanding to mitigate and prevent disease, and to promote health. The goal of digital epidemiology is exactly the same…Digital epidemiology is epidemiology that uses data that was generated outside the public health system, i.e. with data that was not generated with the primary purpose of doing epidemiology.

Epidemiology, in general, is “the science of occurrence of diseases in human populations. Disease occurrence is measured and related to different characteristics of individuals or their environments”. Although clinical trials are sometimes considered a subset of epidemiology, we restrict our discussion here to observational studies of patients who receive their implants as a result of routine care.

The epidemiology of implanted devices includes the following: (1) Descriptions of patients with implants in terms of demographics, underlying disease, and concurrent disease. (2) Assessment of efficacy and safety of the implant. (3) Comparison of treatments.

The concepts mentioned in the definitions of subspecialty of epidemiology are similar to those found for general epidemiology. Some authors did not provided general definition of epidemiology [54–58], nor specific definition to subspecialty [59–61].

Others took up the main concepts of epidemiology and adapted them to their subspecialty. In this respect, the definition of the epidemiology of obesity speaks for itself: it contains virtually all concepts: “causes and consequences”; “human populations”; “the distributions, patterns, and dynamics”; “the determinants”; “to prevent and control”; “health conditions”. This definition also mentions a specificity of the subspecialty: “the development and validation of body composition measurement methods used in epidemiologic studies”.

Others referred to authoritative epidemiological literature or to WHO. For this case, the definitions of Last, 1995 and 2001 [43, 62] were most often cited (6 times out of 24 definitions), WHO (1994), Rothman and Greenland, 2008 [24], Susser, 1973 [63] and Wikipedia being quoted once. Different subspecialties referred to the definition of Last: oncology [64], psychiatry [65], genetic epidemiology [66, 67], reproduction [68].

Finally, others Other authors proposed a pure and simple application of the general definitions of epidemiology to their subspecialty [64, 65]. Thus, Dos Santos after recalling Last's definition of epidemiology proposed a definition of cancer epidemiology and stated: “Therefore, this definition is as valid to cancer epidemiology as it is to epidemiology in general” [64]. Tsuang defined psychiatric epidemiology as follow “Psychiatric epidemiology is simply the epidemiology of psychiatric disorders–no more, no less [65].

Some authors provided definitions by highlighting differences. This is the case, for example, for genetic epidemiology. Austin stated “Genetic epidemiology differs from epidemiology by its explicit consideration of genetic factors and family resemblance” [58].

### Veterinary medicine

Table 6 shows the frequencies of appearance of terms in definitions of epidemiology from 1977 to 2015, for veterinary medicine.

**Table 6:** Definitions of epidemiology from the period 1977–2015. Veterinary medicine.

AUTHOR (Reference)	DATE	DEFINITION
**Schwabe CW et al. [73]**	1977	The study of the health status of populations” (Schwabe et al., 1977). Quoted reference: [73]
**Martin SW et al. [146]**	1987	Epidemiology may be defined as the study of the patterns of disease that exist under field conditions. More specifically, epidemiology is the study of the frequency, distribution, and determinants of health and disease in populations.
**Toma B [147, 148]**	1999	The study of the health status of populations.
**Slater MR [149]**	2003	What is epidemiology? Most simply, it is the study of diseases and health in populations. Diseases include not only the classic infectious diseases and disease outbreaks but also chronic diseases like arthritis, cancer, and renal failure. Also included are injuries and exposures to environmental contaminants. Factors that maintain good health and quality of life are also within the purview of epidemiology. In this context, questions about the causes of animals being relinquished to animal shelters or about animal well-being in long-term kennel settings may also be addressed by epidemiologists.
**Smith RD [147]**	2005	Over the years there have been many definitions of epidemiology. Some definitions follow: 1. “The study of the distribution and determinants of disease frequency in man” (MacMahon and Pugh, 1970). 2. “The study of the patterns of disease” (Halpin, 1975). 3. “The study of the health status of populations” (Schwabe et al., 1977). 4. “Epidemiology is nothing more than ecology with a medical and mathematical flavor” (Norman D. Levine, 1990, personal communication). 5. “The study of the distribution and determinants of health-related states or events in specified populations and the application of this study to control of health problems” (Stedman’s Medical Dictionary, 2000). Quoted references:[69, 73, 150]
**Pfeiffer D [71]**	2009	…the science of veterinary epidemiology deals with the investigation of these determinants of disease distribution in animal populations. Productivity and welfare of animals may also be outcomes of interest since disease will usually impact on both, and they may indeed often be what stakeholders will have as their primary focus.
**Dohoo I et al. [70]**	2009	Epidemiology is largely concerned with disease prevention and therefore, with the "succession of events which result in the exposure of specific types of individual to specific types of environment" (ie exposures) (MacMahon & Pugh, 1970). Thus, epidemiologists strive to identify these exposures and evaluate their associations with various outcomes of interest (eg health, welfare, productivity) so as to improve the lives of animals and their keepers. … associations which are likely to be causal in nature and which, once identified, we can take advantage of to improve the health, welfare and productivity of animals and the quality and safety of foods derived from them. Quoted reference: [69]
**Thrusfield M [72]**	2013	Epidemiology is the study of disease in populations and of factors that determine its occurrence; the key word being populations. Veterinary epidemiology additionally includes investigation and assessment of others health-related events, notably productivity.
**Sergeant et al. [151]**	2015	Epidemiology is the study of patterns and causes of disease in populations. Understanding these issues will in turn contribute to identification of options for control and prevention of diseases. As its simplest, epidemiology is about supporting better decision making to ensure appropriate response or preventative measure for population health.

A total of 9 definitions were selected

Again, the concepts mentioned in the definitions of veterinary epidemiology are similar to those found for human epidemiology, with several authors using definitions proposed in human medicine and quoting for example MacMahon and Plug [69].

One notable concept, specific to veterinary medicine, is productivity which is mentioned by two authors [70–72].

### General epidemiology (websites)

The definitions of epidemiology are given in Table 7 and are classified according to the nature of the website: websites of international organizations, WHO, CDC, FAO (for veterinary medicine); national health institutes, academies of sciences …; public health schools; medical journals (BMJ); nonprofit organizations; Wikipedia; For-profit organizations; non-medical dictionaries online.

The distribution of concepts, by order of frequency, is the following: health (12/30); population (18/30); disease / illness (27/30); study (19/30); distribution (16/30). This distribution is almost that found with the definitions of epidemiology provided by books.

**Table 7:** Definitions of epidemiology available on websites.

AUTHOR and url	DEFINITION	Comments
**WHO** http://www.who.int/topics/epidemiology/en/	Epidemiology is the study of the distribution and determinants of health-related states or events (including disease), and the application of this study to the control of diseases and other health problems. Various methods can be used to carry out epidemiological investigations: surveillance and descriptive studies can be used to study distribution; analytical studies are used to study determinants.	
**CDC** https://www.cdc.gov/ophss/csels/dsepd/ss1978/lesson1/section1.html	Epidemiology is the study of the distribution and determinants of health-related states or events in specified populations, and the application of this study to the control of health problems.	Online version of a printed textbook. Dicker, 2006 [152] Definition based on Last, 2001 [43].
**CDC** Excellence in Curriculum Innovation through Teaching Epidemiology (EXCITE) CDC a self-study course https://www.cdc.gov/careerpaths/k12teacherroadmap/index.html	Epidemiology is the study of the distribution and determinants of health-related states or events in specified populations, and the application of this study to the control of health problems.(1) (1) Last, 2001	Quoted by Dicker, 2006 [152] And based on Last, 2001 [43].
**Australasian Epidemiological Association** https://aea.asn.au/about/epidemiology/what-is-epidemiology	…The study of diseases or other health related events in populations. Epidemiology has three main aims: • To describe disease and other health related event patterns in human populations. • To identify the causes of diseases and other health related events (also known as aetiology). • To provide data essential for the management, evaluation and planning of services for the prevention, control and treatment of disease and other health related events.	
**FAO** (**Food and Agriculture Organization of the United Nations**) http://www.fao.org/wairdocs/ilri/x5436e/x5436e04.htm	… A useful general definition is that given by Schwabe et al (1977), which defines epidemiology as the study of disease in populations.	Based on Schwabe, 1977 [73].
**National Institute on Deafness and Other Communication Disorders** https://www.nidcd.nih.gov/health/statistics/what-epidemiology	Epidemiology is the branch of medical science that investigates all the factors that determine the presence or absence of diseases and disorders. Epidemiological research helps us to understand how many people have a disease or disorder, if those numbers are changing, and how the disorder affects our society and our economy.	“Last Updated Date: September 13, 2011”
**The National Academies of Sciences, Engineering, and Medicine** https://www.nap.edu/read/5804/chapter/2#3	Modern epidemiology, the study of disease patterns in populations, encompasses a broad array of subject matter, including subspecialties that concentrate on such domains as clinical trials of pharmaceutical agents; such outcomes as reproductive and developmental effects, infectious diseases, and chronic diseases; such risk factors as occupation, nutrition, and alcoholism; and special populations	
**National Research Council (US). Committee on Environmental Epidemiology, National Research Council (US). Commission on Life Sciences. Environmental Epidemiology** http://www.ncbi.nlm.nih.gov/books/NBK233638/	Modern epidemiology, the study of disease patterns in populations, encompasses a broad array of subject matter, including subspecialties that concentrate on such domains as clinical trials of pharmaceutical agents; such outcomes as reproductive and developmental effects, infectious diseases, and chronic diseases; such risk factors as occupation, nutrition, and alcoholism; and special populations	Date of publication: 1997
**Encyclopedia of occupational health and safety. 4th edition** International Labour Office. http://www.ilocis.org/documents/chpt28e.htm Chapter 28—Epidemiology and Statistics Epidemiological method applied to occupational health and safety. Franco Merletti, Colin L. Soskolne and Paolo Vineis	Several operational definitions of epidemiology have been suggested. The simplest is that epidemiology is the study of the occurrence of disease or other health-related characteristics in human and in animal populations. Epidemiologists study not only the frequency of disease, but whether the frequency differs across groups of people; i.e., they study the cause-effect relationship between exposure and illness. Diseases do not occur at random; they have causes—quite often man-made causes—which are avoidable…	
**School of Public Health**. **University of Alabama at Birmingham**. http://www.soph.uab.edu/epi/academics/studenthandbook/what	Many definitions have been proposed; here are two that capture the underlying principles and the public health spirit of epidemiology: “Epidemiology is the study of the distribution and determinants of health-related states or events in specified populations, and the application of this study to the control of health problems.” (2). “Epidemiology is the study of the distribution and determinants of disease frequency in man.” (3). (2) Last JM, 1995. (3) MacMahon & Pugh, 1970	Definition Based on Last, 1995 [62] and MacMahon, 1970[69].
**School of Public Health. Boston University** http://sphweb.bumc.bu.edu/otlt/mph-modules/ep/ep713_history/EP713_History9.html#headingtaglink_3	Epidemiology is the study of the distribution and determinants of disease frequency in human populations. Epidemiology focuses on making comparisons in order to establish cause-effect relationships, evaluate information, and make good decisions that will improve outcomes.	
**Public Health Social Work Maternal and Child Health Leadership Training Program** **University of North Carolina at Chapel Hill** https://ssw.unc.edu/mch/node/148	Epidemiology is the “study of the distribution and determinants of health-related states or events (including disease), and the application of this study to the control of diseases and other health problems” (World Health Organization, 2011). … Epidemiology examines how health conditions are distributed among a population and seeks to understand the risks or causes associated with those conditions.	Based on WHO (2011) definition
**BMJ** http://www.bmj.com/about-bmj/resources-readers/publications/epidemiology-uninitiated/1-what-epidemiology	Epidemiology is the study of how often diseases occur in different groups of people and why. Epidemiological information is used to plan and evaluate strategies to prevent illness and as a guide to the management of patients in whom disease has already developed.	
**School of Public Health** **Colorado** http://www.ucdenver.edu/academics/colleges/PublicHealth/Academics/departments/Epidemiology/About/Pages/Learn.aspx	Epidemiology is the study of patterns of disease and injury in human populations and the application of this study to the control of health problems. Epidemiologists study the variation of disease in relation to age, sex, race, occupational and social characteristics, place of residence, susceptibility, exposure to specific agents or other pertinent characteristics	
**Fulton county, Georgia** http://www.fultoncountyga.gov/dhw-epidemiology/8261-what-is-epidemiology	Epidemiology is a branch of science that deals with the incidence, distribution and control of disease in a population. It is a core public health science. Epidemiologists monitor health trends and statistics to identify groups of people who are affected by various diseases. In addition, epidemiologists investigate cases of disease to determine the source, modes of transmission, and risk factors for disease.	
**Epidemiology Education Movement** http://www.epiedmovement.org/BM3.html	Epidemiology is a fundamental population science and tries to answer questions about health-related behaviors and outcomes in populations. Epidemiologists study how health and disease are distributed in populations and the factors that influence or determine those distributions.	
**People's Epidemiology Library** http://www.epidemiology.ch/history/PeopleEpidemiologyLibrary.html	Epidemiology is the science of counting health-related events and comparing these counts over time, place and people. Health-related events are the diseases themselves and their causes.	
**Merck manual. Veterinary** http://www.merckvetmanual.com/public-health/public-health-primer/basic-principles-of-epidemiology	The definition of epidemiology is “the study of disease in populations and of factors that determine its occurrence over time.” The purpose is to describe and identify opportunities for intervention. Epidemiology is concerned with the distribution and determinants of health and disease, morbidity, injury, disability, and mortality in populations. For veterinary epidemiology, this intervention is to enhance not only health but also productivity.	Productivity is taken into account
**Capella University** Minneapolis (Profit Institution) https://www.capella.edu/blogs/cublog/what-is-epidemiology/	We often think about epidemiology in terms of disease; however, it is really the study of how to keep the population healthy. A large of part of epidemiology is determining the cause of disease, providing education about it, and creating plans to prevent and mitigate widespread illnesses	
**Wikipedia** https://en.wikipedia.org/wiki/Epidemiology	Epidemiology is the study and analysis of the patterns, causes, and effects of health and disease conditions in defined populations. It is the cornerstone of public health, and shapes policy decisions and evidence-based practice by identifying risk factors for disease and targets for preventive healthcare.	
**https://www.kullabs.com/classes/subjects/units/lessons/notes/note-detail/3453**	Epidemiology is the study and analysis of the patterns, causes and effects of health and disease conditions have defined the population. It is derived from the word epidemic (epi -among; demos-people; logos -study). "The study of distribution and determinants of health-related or events in specified populations and the application of this study to the control of health problems."	Based on WHO, CDC and Wikipedia Following links are provided by this internet site are: •www.who.int/topics/epidemiology/en/ •www.who.int/mediacentre/factsheets/fs355/en/ •www.cdc.gov/ophss/csels/…/section1.htm •www.cdc.gov/…/data_stats/glossary.html •https://en.wikipedia.org/wiki/Non-communicable_disease
**healthcare-management** http://www.healthcare-management-degree.net/faq/what-is-epidemiology-and-what-does-an-epidemiologist-do / Degree Guide	Epidemiology is a field where trained epidemiologists study patterns of frequency and the causes and effects of diseases in human populations	
**Business Dictionary** http://www.businessdictionary.com/definition/epidemiology.html	Study of distribution and pattern of diseases in a population to determine or trace the circumstances or events causing them. This information is used by public health authorities in designing appropriate policies and interventions to protect the population.	
**Cambrigde Dictionary** http://dictionary.cambridge.org/dictionary/english/epidemiology	The scientific study of diseases and how they are found, spread, andcontrolled in groups of people	
**Collins Dictionary** https://www.collinsdictionary.com/dictionary/english/epidemiology	Epidemiology is a branch of medicine that is concerned with the occurrence, distribution, and control of disease.	
**Dictionary.com** http://www.dictionary.com/browse/epidemiology	The branch of medicine dealing with the incidence and prevalence of disease in large populations and with detection of the source and cause of epidemics of infectious disease.	
**The Free Dictionary** http://www.thefreedictionary.com/Epidemiolgy	Based on several dictionaries: American Heritage, Collins, Webster…	
**The Law Dictionary** http://thelawdictionary.org/epidemiology/	Study of distribution, pattern, and cause of diseases in a specific population. The objective is to determine and trace events and reasons causing epidemic-like situations. Resulting information leads to more effective plans, policies, and containments protecting the population by public health authorities.	
**Oxford Dictionary** https://en.oxforddictionaries.com/definition/epidemiology	The branch of medicine which deals with the incidence, distribution, and possible control of diseases and other factors relating to health.	
**Webster Dictionary** http://www.webster-dictionary.org/definition/epidemiology	That branch of medicine which studies the incidence and distribution of disease in a population, and uses such information to find the causes, modes of transmission, and methods for control of disease.	

The majority of websites offer definitions of epidemiology already known, but does not cite the authors.

CDC and FAO based their definition on Last, 2001 [43] and Schwabe, 1977 [73], respectively.

Public health schools do not provided the source of their definition of epidemiology except one (University of Alabama) with Last, 2001 [43] and MacMahon, 1970 [69] being quoted.

Few websites mentioned the date of the last update (e.g. National Institute on Deafness and Other Communication Disorders).

## Discussion

This study allowed us to identify, synthesize and analyse the definitions of epidemiology from the period 1978–2017. This evolution is almost absent from books on epidemiology and deserved to be presented. Even though many other fields of epidemiology have grown, only definitions of epidemiology were studied. A significant number of definitions of epidemiology were found despite the short period of study and the inclusion criteria. However, this number must be related to the increase in the number of publications in epidemiology.

Many of the books consulted and the definitions of epidemiology they contained summarized definitions that were already known [74] or definitions of other authors [75] or proposed mnemonics tools "the 3-D definition of epidemiology" [76, 77].

In summary, 69 definitions of epidemiology were retained from 1978 to 2017. All of these came from epidemiology textbooks or published articles. Only one definition (WHO) was not associated with a date of appearance [32]. The fact that this definition was not cited by Lilienfeld was enough to keep it in this study.

The evolution of the content of definitions of epidemiology was studied on the basis of terms and concepts identified by Evans [3] and those identified by us.

The definitions from 1978 to 2017 helped to highlight 20 terms and concepts related to epidemiology. A great majority of concepts were either identified by Evans [3] or present in definitions used by Lilienfeld [1]. Among the terms identified, only three (“*problems*”, “*knowledge*” and “*public health*”) were not present in Evan’s work. Five terms were present in more than 50% of definitions from 1978 to 2017: “*population*”, “*study*”, “*disease*”, “*health*” and “*distribution*”. These five terms can summarize epidemiology as being the study of the distribution of disease and health in the population. The term “*study*” may not seem specific enough for epidemiology. But this term is linked to the concept of “*science*”. Epidemiology was considered a “*science*” during both periods. In fact, the term “*science*” was used in more than 15% of definitions for the two periods.

Several developments have occurred. (i) the emergence of the term “*problem*” in the definitions of epidemiology; (ii) strengthening of the terms “*control*” and *“health”* already used; (iii) other terms and concepts (“*infectious* diseases”, “*mass phenomenon*”) identified by Evans [3] were no longer used in definitions from 1978 to 2017. But these terms were rare in the definitions used by Lilienfeld: 4/23 (17%), 1/23 (4%), 3/23 (13%) and 3/23 (13%), respectively.

The prevalence and distribution of diseases, infectious or not, also influence epidemiology. Thus, in low-income countries, new infectious diseases have emerged such as HIV infection. Nevertheless, the prevalence of these infectious diseases has declined over time. In low-income countries, infectious diseases are still present [78], but there is an increase in the incidence of chronic diseases [79]. Epidemiological definitions have evolved in this direction, with a decrease in the presence of infectious disease terms.

Indeed, for definitions from 1978 to 2017, only one definition associated the terms “epidemiology” and “epidemic” [80]. Currently, the term “epidemic” is associated with both infectious disease and the growing prevalence and incidence of disease states in the population, for example, obesity (obesity epidemic) [81]. Thus, the concept of “disease” used in epidemiology is no longer limited to infectious diseases.

Epidemiology is currently associated with the study of disease, but also more broadly, with the study of health phenomena. In 23% (16/69) of the definitions retrieved, the concepts of “*disease*” and the concept of “*health*” were related. The term “disease” has a rather negative connotation, while the term “health” has a positive connotation. They reflect two opposing views of the same phenomenon. Some definitions suggested this phenomenon was dynamic and not static and evolved in the population, in space and in time [3, 33, 38, 82, 83].

In the definitions from 1978 to 2017, the link between epidemiology and public health was rare [16, 18, 20, 44, 84]. However, these definitions describe epidemiology as an essential part of public health. Other developments, nonetheless, were related to public health, like the expansion of the field of epidemiology to areas such as health promotion [18] and the use of statistical tools [3, 30, 32, 52].

Finally, 69 definitions (general epidemiology) were analyzed. This is greater than the number of definitions used by Lilienfeld. The fact that the definitions retained came from different sources, both grey and published literature, suggests that the definitions retained are representative of the available definitions of epidemiology, thus limiting the risk of selection bias. The evolution of the content of definitions of epidemiology was studied by using the concepts identified by Evans [3] and those identified by us. The comparison with the concepts cited by Evans allowed an objective analysis of the evolution of the content of definitions of epidemiology from 1978 to 2017. The statistical analysis revealed the differences observed between the two sets of definitions of epidemiology, even if the definitions of epidemiology from 1978 to 2017 were not exhaustively from this period.

The analysis of the evolution of the definitions of human epidemiology by subspecialty and veterinary epidemiology was not possible, due to the low number of definitions.

The number of subspecialties of epidemiology increases from year to year and almost all fields of medicine are concerned ([85]). As early as 2007, Lillienfeld already raised the question of the general epidemiologist [86]. As for general epidemiology, they are several definitions for each subspecialty. Austin [58], quoting Khoury's book published in 1993 [87], mentions that there were at least eight different definitions of genetic epidemiology at that time.

This increase in the number of definitions of epidemiological subspecialties can be linked to several factors including technological advances. The significant development of data analysis in epidemiology (epidemiological methods) could be related to the emergence of informatics. Molecular and genetic epidemiology is also undoubtedly linked to major advances in molecular and genomic biology [88]. Advances in genome sequencing techniques have led to a better understanding of the genetic determinants of disease occurrence. They have therefore promoted the emergence of genetic epidemiology as a subspecialty of epidemiology [89], with a specific definition [90].

The prevalence of some cancers has also changed over time. Epidemiology has made it possible to identify some risk factors related to the environment and lifestyles. Nevertheless, the etiology of many types of cancer is still poorly understood. Indeed, it is generally accepted that most cancers result from the combined effects of environmental and genetic factors and that only a few cancers are only "of genetic origin" [91]. The integration of molecular techniques into epidemiological studies has also led to the emergence of molecular epidemiology [92], with a definition proposed by Schulte et al. in 2012 [57].

Ecology and environment are a concern of human populations. The same applies to their impact on health. The emergence of environmental epidemiology is a particular reflection of this. The consideration of environment in the definition of epidemiology appeared in the 2000s, with the definition of Gerstman [30], which includes the term "modern" (with reference to old definitions). In previous years, only Cole, 1979 [82] refers to the environment in his definition of epidemiology.

The digital revolution, linked to the rapid and unprecedented increase in the availability of data from various digital sources, has created new opportunities for collecting and analyzing data produced outside the health system. This led Salathé et al. [93] to introduce the term "digital epidemiology" and to propose a definition in 2018 [94]. Some tools such as Google Trends are available and their use has led to mixed results [95].

The use of medical devices, whether implanted or not, has become crucial in many medical specialties for the diagnosis and treatment of certain diseases. Although their use is frequently beneficial to the patient, side effects may occur sometimes (e. g. infection on a vascular catheter). Monitoring these side effects and studying the risk factors for their occurrence is a necessity that has led to the individualization of the epidemiology of medical devices, with a specific definition [96, 97]. The methodological aspects specific to this field of epidemiology (definition of "real" exposure; choice of short- or long-term assessment criteria; various biases) were reported by Jalbert et al. [98].

Faced with the numerous specialties and the numerous books about on the topics, we had to make a selection. We recall that the main objective was general epidemiology, the description of the definitions of subspecialties of epidemiology being a complement.

The concepts found in the definitions of subspecialties of human epidemiology and those of veterinary epidemiology are generally the same. Consideration of economic aspects (productivity) is a specific feature of veterinary epidemiology.

The concepts found in the definitions of human epidemiology available on the websites are generally the same as those contained in the books or articles. Nevertheless, bibliographic references are often unavailable and access of information may change over time, with the disappearance of pages [99] or the existence of dead links ("error 404"…). The posting date is also rarely available.

Publications about the definitions of epidemiology exist. The fields and the aim are for example: introductory epidemiology textbooks [10]; description of definitions epidemiology over 50 years [9]; epidemiology and the methods needed for public health assessments [100]; evolution of epidemiological methods and concepts [11]. But our review examines the evolution of definitions of epidemiology, taking into account more than 100 definitions of epidemiology retrieved from books or articles, introductory texts or not, general epidemiology or subspecialties, and a non-temporal description and analysis of the definitions of veterinary medicine. We also describe 29 definitions of epidemiology retrieved from websites.

This study has several limitations. Only English definitions were retained. National definitions of epidemiology not translated into English were eliminated [101]. These definitions may have contained terms and concept related to epidemiology that were different from those in the definitions retained. But the probability that we have missed an important concept seems quite low. The terms and concepts identified depend on the content of the definitions selected. The definitions of epidemiology available in online dictionaries and other media were easy to find but the quality of the definitions was heterogeneous. To determine the quality of definitions, quality criteria need to be used. For the definitions from 1978 to 2017, the quality criteria may seems subjective. Moreover, no weights were assigned to definitions. The choice of weighting criteria may be subjective.

The purpose of the study was to identify concepts related to epidemiology and not to prioritize them. Given the variety of definitions, the fact that the method used to identify terms and concepts was easy to use and the fact that the majority of terms and concepts were present in more than 10% of definitions suggests that for the definitions retained all of the terms and concepts related to epidemiology were identified.

For future studies, we propose some weighting criteria such as inverse-variance weighting (meta-analysis…) which are widely accepted. We also propose that some of the following criteria be taken into account in the weighting grid: the number of concepts in the definition; the book type (collective dictionary as those of Last/ Porta; advanced texts; introductory texts).

## Conclusions

In summary, this work led to a synthesis of different concepts related to epidemiology proposed during the period 1978–2017 and highlighted the evolution of the content of definitions of epidemiology over time. Most of the terms and concepts identified by us had already been used in the definitions of Lilienfeld while several terms and concepts identified by Evans were no longer used in definitions from 1978 to 2017. Increased usage of the terms “control” and “health” was found in definitions of epidemiology from 1978 to 2017. A thematic analysis of definitions of epidemiology could be conducted to complete this study, in order to improve our understanding of the changes observed.
